# Inhibition of BRD4 triggers cellular senescence through suppressing aurora kinases in oesophageal cancer cells

**DOI:** 10.1111/jcmm.15901

**Published:** 2020-09-20

**Authors:** Jian‐Ling Xu, Ya‐Jiao Yuan, Jiao Lv, Di Qi, Meng‐Di Wu, Jing Lan, Sheng‐Nan Liu, Yong Yang, Jing Zhai, Han‐Ming Jiang

**Affiliations:** ^1^ Department of Biochemistry School of Basic Medical Sciences Shandong First Medical University & Shandong Academy of Medical Sciences Jinan China; ^2^ Department of Clinical Laboratory People’s Hospital of Jimo District Qingdao China; ^3^ Department of Clinical Laboratory The Second Affiliated Hospital of Shandong First Medical University Taian China

**Keywords:** aurora kinase, BRD4, cellular senescence, oesophageal cancer

## Abstract

Oesophageal cancer is one of the most frequent solid malignancies and the leading cause of cancer‐related death around the world. It is urgent to develop novel therapy strategies to improve patient outcomes. Acetylation modification of histones has been extensively studied in epigenetics. BRD4, a reader of acetylated histone and non‐histone proteins, has involved in tumorigenesis. It has emerged as a promising target for cancer therapy. BRD4 inhibitors, such as JQ1, have exerted efficacious anti‐proliferation activities in diverse cancers. However, the effects of JQ1 on oesophageal cancer are still not fully described. Here, we demonstrate that JQ1 suppresses cell growth and triggers cellular senescence in KYSE450 cells. Mechanistically, JQ1 up‐regulates p21 level and decreases cyclin D1 resulting in G1 cycle arrest. The inhibitory effects of JQ1 on KYSE450 cells are independent on apoptosis. It activates cellular senescence by increasing SA‐β‐gal activity. BRD4 knockdown by shRNA recapitulates cellular senescence. We also display that administration of JQ1 decreases recruitment of BRD4 on the promoter of aurora kinases A and B. Inhibitors targeting at AURKA/B phenocopy JQ1 treatment in KYSE450 cells. These results identify a novel action manner of BRD4 in oesophageal cancer, which strengthens JQ1 as a candidate drug in oesophageal cancer chemotherapy.

## 1 INTRODUCTION

Oesophageal cancer is one of deadliest cancer and ranks seventh incidence and sixth mortality globally.^1^ Oesophageal carcinoma is mainly classified into two major subtypes, oesophageal adenocarcinoma (EAC) and oesophageal squamous cell carcinoma (ESCC). ESCC is more common in the Asian, African and South American, while EAC occurs predominantly in North America and Europe.^2^, ^3^ Oesophageal carcinoma is still the main disease burden of the local residents.^4^ Currently, treatments employed for oesophageal carcinoma mainly include radiation therapy, chemotherapy, surgical treatment and their combinations.^5^, ^6^, ^7^ However, there are few precise approaches for the treatment of oesophageal carcinoma, and the 5‐year survival rate of oesophageal cancer remains dismal.^8^, ^9^ It is urgent to identify more effective and targeted strategies for these cancers treatment.

Epigenetics refers to the heritable changes occurring in the activities of genes without undergoing changes in the nucleotide sequences. Epigenetic regulation takes the important part in controlling cell identity and functions. Disorder of epigenetics is closely associated with human diseases, such as cancer. Histone modification is an important part of epigenetics, of which acetylation is the earliest type of mechanism discovered and studied in detail. Histone acetylations can be ‘read out’ by specific proteins known as epigenetic readers. Small molecules targeting epigenetic readers have emerged as novel strategies in cancer therapy. BRD4 is a member of the bromodomain and extraterminal domain (BET) family containing two bromodomains and one super‐terminal structure, and is an important reader of Histone acetylation.^10^, ^11^, ^12^ Numerous evidences indicate that BRD4 plays an important role in the progress of haematological malignancies as well as solid tumours, indicating as a potential target for cancer therapy. BRD4 mainly binds to histones acetylated by the promoters, enhancers or super‐enhancers of oncogenes, and then mediates their expressions to promote the occurrence and progression of tumours, such as multiple myeloma, acute myeloid leukaemia and prostate cancer.^13^, ^14^, ^15^ Knockdown of BRD4 or BET inhibitors are known to demonstrate strong antitumour activity by inducing cell cycle arrest, apoptosis， differentiation and metastasis of various cancer cells. However, the effects of BRD4 inhibitor on the proliferation and its underlying mechanisms in oesophageal carcinoma are not fully discovered. In this study, we demonstrate that administration of JQ1 to oesophageal carcinoma cells disrupts their growth, especially for KYSE450 cells. Remarkably, inhibition of BRD4 induces cellular senescence instead of apoptosis. Mechanistically, JQ1 reduces BRD4 recruitment to the promoter of Aura A and Aura B, which in turn induces cellular senescence.

## 2 MATERIALS AND METHODS

### 2.1 Cell lines and reagents

All cell lines used in this study were purchased from the cell bank of Chinese Academy of Sciences. KYSE450 cells were cultured in the minimum essential medium (MEM). TE10 cells were maintained in Dulbecco's modified Eagle’s medium with high glucose concentrations. KYSE180 and KYSE510 cells were maintained in RPMI‐1640. All media were supplemented with 10% foetal bovine serum and 1% penicillin/streptomycin (Solarbio). BRD4 inhibitor JQ1, Aura A inhibitor alisertib and Aura B inhibitor barasertib were purchased from Selleckchem. They were dissolved in dimethyl sulphoxide (DMSO) to an indicated concentration stored at −20°C. Antibodies against BRD4, p21, cyclin D1, Aura A and Aura B were from Abcam. Cleaved PARP and cleaved caspase‐3 antibodies were purchased from Cell Signaling Technology; BRD4 antibody for the chromatin immunoprecipitation (ChIP) was from Bethyl Laboratories. GAPDH antibody was from Santa Cruz Biotechnology. Lentiviral shBRD4 plasmid was purchased from Genechem. Lipofectamine^®^ RNAiMAX Transfection Reagent was from Invitrogen. Cell Counting Kit‐8 (CCK‐8) was from Beyotime.

### 2.2 Cell viability assays

To investigate the anti‐proliferation of BRD4 to oesophageal cancer cells, KYSE510, KYSE180, KYSE450 and TE10 cells were seeded in 96‐well plates at 2.5 × 10^3^ cells/well overnight, and then the cells were treated with different concentration of JQ1. After 72 hours, the cells were incubated with 10 μL CCK‐8 (5 mg/mL) for 1 hour. Then, the plates were measured at 450 nm by microplate reader (PE Enspire).The OD values of wells containing only CCK‐8 were regarded as the A value (background value), based on which the cell viability was calculated as follows: Cell viability = (OD values of JQ1‐treated groups − A value)/(OD value of control group − A value) × 100%. The half‐maximal inhibitory concentration (IC50) values were calculated using a GraphPad Prism 7.0. Each experiment was repeated at least three times.

### 2.3 Cell cycle analysis

All cells used to perform experiments were in the logarithmic phase. KYSE450 cells were seeded in a six‐well plate at 2.0 × 10^5^ cells/well and incubated for 24 hours. Then, the cells were treated with JQ1 at 0.25, 0.5 and 1 μmol/L for 24 hours. For cell cycle assays, the harvested cells were digested and washed twice with cold phosphate buffer saline (PBS), and the cell pellets were suspended in 70% pre‐cold ethanol at 4°C overnight. The fixed cells were washed twice with cold PBS and then stained in 500 μL PBS containing 50 mg/L RNase A and 50 mg/L propidium iodide (PI) at 37°C for 30 minutes in the dark. The cells were filtered and analysed on flow cytometry.

### 2.4 Apoptosis assays

KYSE450 cells were seeded in a six‐well plate at concentrations of 2.0 × 10^5^ cells/well overnight. After treatment with indicated JQ1 for 72 hours, the cells were harvested and incubated with in FITC Annexin V and Propidium Iodide Staining Solution (BD Pharmingen) for 15 minutes at room temperature in the dark. Apoptosis was investigated on flow cytometry.

### 2.5 Protein isolation and Western blot analysis

After different treatments, KYSE450 cells were collected and the cell lysates were prepared in lysis buffer containing fresh protease inhibitor cocktail (Solarbio). The samples were mixed with loading buffer and boiled for 5 minutes at 95°C. Equal amounts of proteins were used to SDS‐PAGE and transferred to a nitrocellulose membrane. After being blocked with 5% non‐fat milk for 60 minutes at room temperature, the membranes were incubated with the diluted primary antibodies at 4°C overnight. Following washing with TBST and TBS, respectively, the membranes were probed with the corresponding horseradish peroxidase–conjugated secondary antibodies at regular temperature for 1 hour. The protein bands were visualized by ECL blotting detection reagents (Millipore). GAPDH (glyceraldehyde‐3‐phosphate dehydrogenase) was selected as the internal reference protein for normalization of the protein amounts.

### 2.6 Reverse transcription PCR (RT‐PCR) and real‐time quantitative PCR (qPCR)

KYSE450 cells were seeded in a six‐well plate at concentrations of 2.0 × 10^5^ cells/well overnight. Then, the cells were treated with JQ1 for 96 hours. Total RNAs were separated from JQ1‐treated cells or control cells by TRIzol Reagent (Ambion) according to the manufacturer’s protocol. cDNA was synthesized by the ReverTra Ace qPCR RT Master Mix with gDNA Remover (TOYOBO) using 1 μg total RNAs. qPCR assays were performed with ChamQTM SYBR^®^ qPCR Master Mix (Vazyme) on Stratagene Mx3005P (Agilent Technologies). The GAPDH gene was used as internal reference.

The sequences of primers were listed as follows:

- P21: Forward, 5′‐TGTCCGTCAGAACCCATGC‐3′,
- Reverse, 5′‐AAAGTCGAAGTTCCATCGCTC‐3′;
- P27: Forward, 5′‐ TAATTGGGGCTCCGGCTAACT‐3′,
- Reverse, 5′‐ TGCAGGTCGCTTCCTTATTC‐3′;
- Cyclin D1: Forward, 5′‐ GCTGCGAAGTGGAAACCATC‐3′,
- Reverse, 5′‐ CCTCCTTCTGCACACATTTGAA‐3′;
- Aura A: Forward, 5′‐ AGGACAAGGGCCTTCTTAGG‐3′;
- Reverse, 5′‐ TAGTGGGTGGGGAGACAGAC‐3′;
- Aura B: Forward, 5′‐ AGCCGTGAGAAGCAGAGAAA‐3′;
- Reverse, 5′‐ ATTGGGGCTAGTGTGCTGAC‐3′;
- GAPDH: Forward, 5′‐ GGAGCGAGATCCCTCCAAAAT‐3′,
- Reverse, 5′‐ GGCTGTTGTCATACTTCTCATGG‐3′.

The program used for amplification: 94°C for 2 minutes, followed by 40 cycles: 94°C for 30 seconds, 60°C for 30 seconds, 72°C for 30 seconds. Levels of gene expression were normalized to GAPDH level, and changes in the transcriptional level were investigated by the ΔΔ*C*_t_ method.

### 2.7 β‐gal staining assay

KYSE450 cells were seeded in a six‐well plate at concentrations of 1.5 × 104 cells/well and incubated for 24 hours. Then, the cells were treated with chemicals or BRD4 shRNA for 6 days. The cells were stained by the Senescence β‐Galactosidase Staining Kit (Cell Signaling Technology) following the manufacturer’s instructions. The positive cells were observed under an optical microscope and photographed.

### 2.8 Chromatin immunoprecipitation and quantitative PCR

KYSE450 was treated with DMSO or JQ1 for 48 hours. Then, the cells were crosslinked with 1% formaldehyde for 10 minutes at room temperature. The crosslinking reaction was terminated by addition of glycine at a final concentration of 1.25M and incubated for 5 minutes at room temperature. The samples were suspended in lysis buffer and sonicated. Chromatin was immunoprecipitated using the BRD4 antibody or control IgG. The specific primers used for quantitative PCR were described previously.^16^

### 2.9 BRD4 shRNA transfection

KYSE450 cells were infected with lentiviral particles harbouring shBRD4 for 2 days with administration of 8 μg/mL polybrene. The cells were incubated with fresh medium for an additional 4 days. The cells were stained by Senescence β‐Galactosidase Staining Kit, and cell lysates were subjected to immunoblot assay.

### 2.10 Statistical analysis

SPSS22.0 (IBM, New York, NY) and the GraphPad Prism 7 software (GraphPad Software Inc., San Diego, CA) were used for statistical analysis. All data were showed a mean ± SD. Statistical analysis was done by two‐tailed Student’s *t* test. A value of *P* < .05 was considered to be statistically significant.

## 3 RESULTS

### 3.1 JQ1 blocks proliferation of oesophageal cancer cells

**Figure 1:**
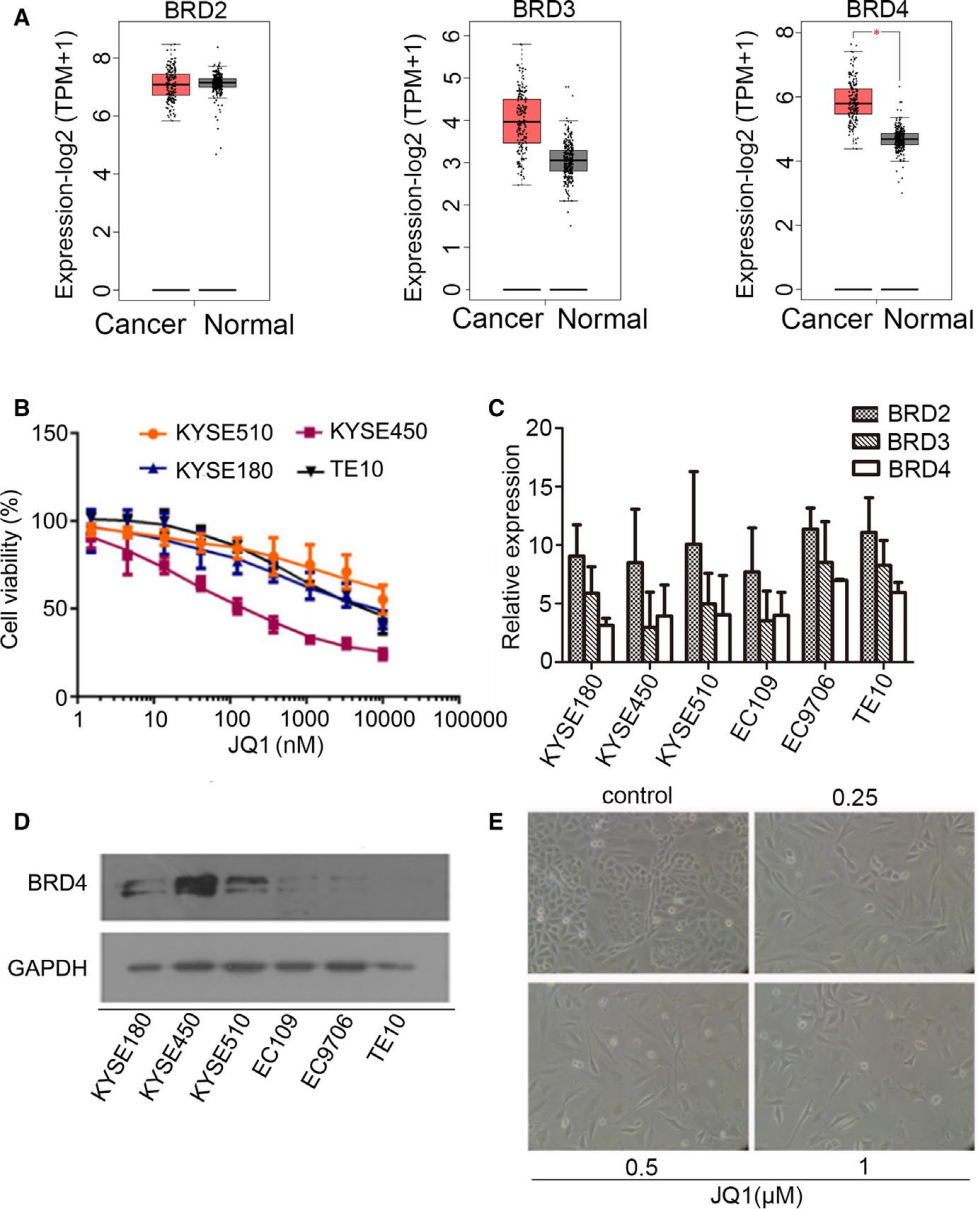
JQ1 inhibited the growth of oesophageal cancer cells. A, Box plots of BRD2, BRD3 and BRD4 expression in tumour tissue (Red, patient number = 182) vs normal tissue (grey, patient number = 286) acquired from GEPIA2 website. B, Four oesophageal cancer cell lines were treated with indicated JQ1 for 72 hours. The cell viability was assayed by CCK‐8. The data represented the mean of triplicate experiments. C, Expression levels of BRD2, BRD3 and BRD4 in oesophageal cancer cell lines using real‐time PCR. The mRNA expressions were normalized by the housekeeping gene GAPDH. The relative expression of *y*‐axis presents the difference of threshold cycles between target transcript and GAPDH. The results were represented as the average of three experiments. D, Protein levels of BRD4 in various oesophageal cancer cell lines using immunoblotting. E, KYSE450 cells were treated with different concentrations of JQ1 and observed under a microscope

BRD4, a member of acetylated histone readers, was overexpressed in some tumours^17^, ^18^ indicating that BRD4 was a promising therapeutic target for cancer therapy. To explore the potential use of JQ1 on oesophageal cancer, we analysed the expression levels of BRD2, BRD3 and BRD4 in oesophageal cancer patients and normal tissues using the data from GEPIA (http://gepia.cancer‐pku.cn/). ^19^ The results showed that the expression of BRD4 highly up‐regulated in oesophageal cancer (Figure 1A), which implied that BRD4 might involve in the growth of oesophageal cancer cells.

To investigate whether administration of JQ1 could affect the proliferation of oesophageal cancer cells, a panel of cell lines was explored to JQ1 for 72 hours. The viability was assessed using the CCK‐8 assay. The results revealed that all the tested tumour cells demonstrated a various growth inhibition with JQ1 treatment in a dose‐dependent pattern. The KYSE450 cells showed more sensitivity to JQ1 treatment with an IC_50_ of approximately 219.5 nmol/L (Figure 1B) due to higher level of BRD4 (Figure 1C,D). After 72‐hour treatment, the morphology of KYSE450 cells changed from round and suspended to flat with loss of stereoscopic appearance and an enlarged nucleus (Figure 1E). Together, these findings revealed that JQ1 could inhibit the growth of oesophageal cancer cells. Higher level of BRD4, more sensitive to JQ1.

### 3.2 JQ1 induced oesophageal cancer cell cycle arrest but not apoptosis

**Figure 2:**
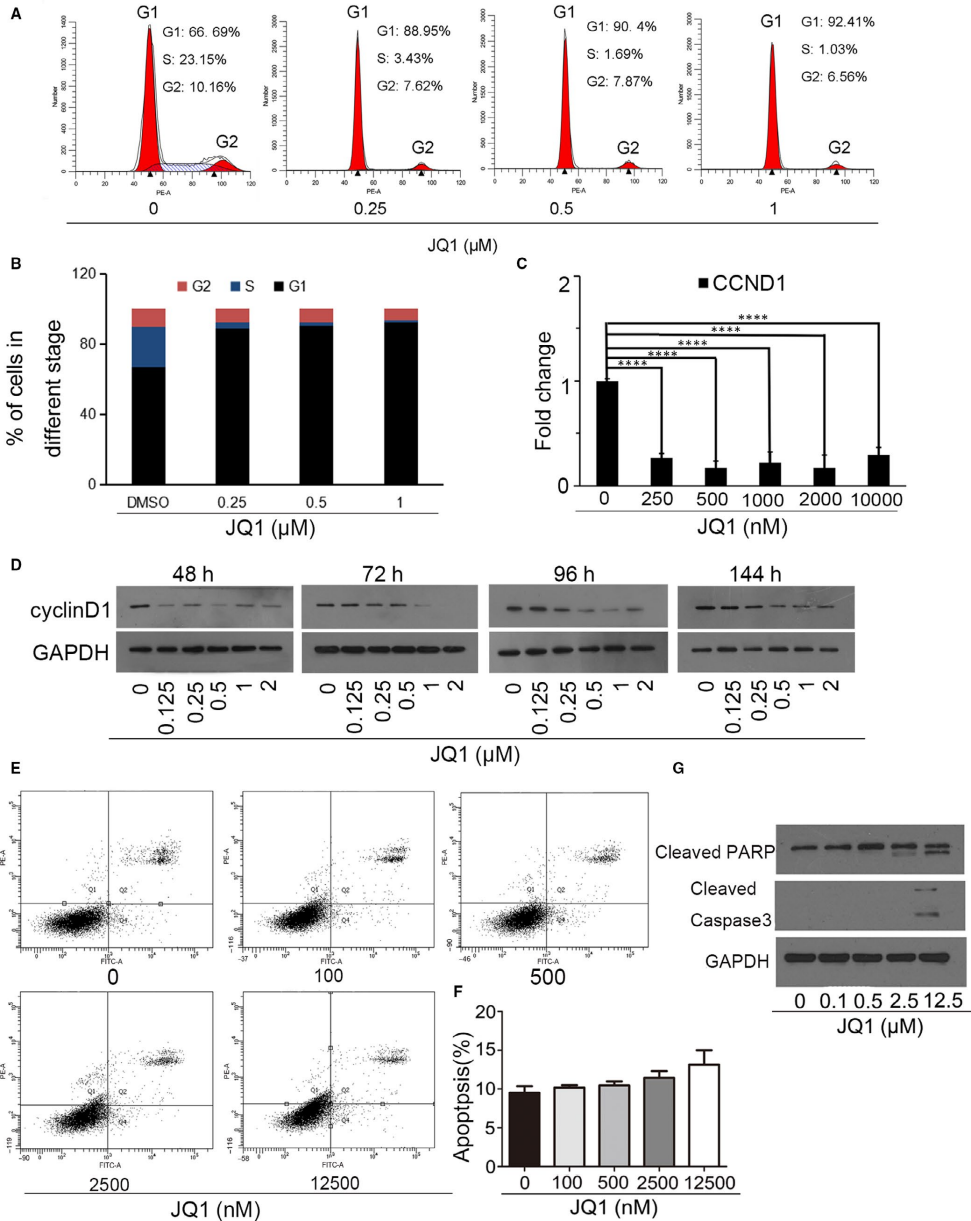
JQ1 arrested cell cycle but not induced apoptosis. A, B, KYSE450 cells were treated with the indicated JQ1 for 24 hours, and the cell cycle distribution was analysed using flow cytometry. C, D, Administration of JQ1 down‐regulated CCND1 expression. ****P ≤ .001. E, F, Apoptosis assay of KYSE450 cells after incubation with vehicle or JQ1 for 72 hours. The data were means ± SD. G, Activation of caspase‐3 and cleavage of PARP treated with indicated JQ1 for 72 hours in KYSE450 cells

To investigate whether the growth inhibition of KYSE450 cells with JQ1 treatment was due to cell cycle arrest, we analysed the cell cycle distribution of KYSE450 cells after JQ1 treatment using flow cytometry and found that the proportion of G1 phase significantly increased with a reduced proportion of S phase (Figure 2A,B). But the percentage of G2 phase cells hardly changed. The results indicated the transition from proliferative phase to a quiescent state after JQ1 treatment in KYSE450 cells. JQ1 treatment time‐ and dose‐dependently reduced expression of CCND1 (Figure 2C,D). Apoptosis has been described as one underlying mechanism of JQ1 on cell proliferation inhibition. To explore the effects of JQ1 on apoptosis, KYSE450 cells were incubated with JQ1 or DMSO for 72 hours, and then apoptotic rate was monitored using Annexin V‐FITC and PI staining. The data showed that the administration of JQ1 slightly induced apoptosis indicating a different cellular response instead of apoptosis (Figure 2E,F) The results of Western blotting also demonstrated that the activation of caspase‐3 and PARP cleavage was observed with high levels of JQ1 treatment (Figure 2G). These data indicated that JQ1 arrested cell cycle at G0/G1 phase in KYSE450 cells, but not induced apoptosis.

### 3.3 Inhibition of BRD4 induced senescence by down‐regulating AURKA and AURKB expression in oesophageal cancer cells

**Figure 3:**
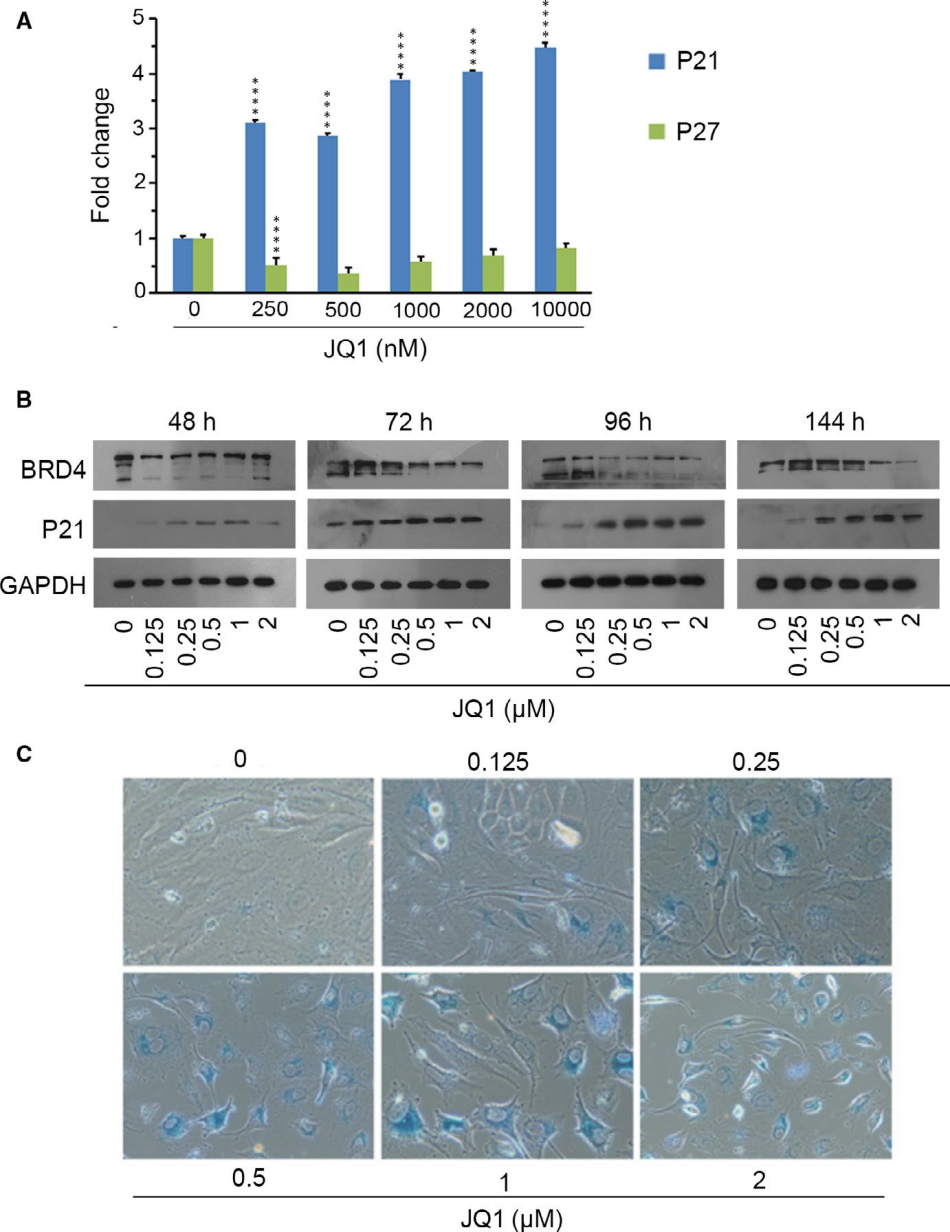
BRD4 inhibition triggered cellular senescence. A, mRNA levels of p21, p27 in KYSE450 cells treated with JQ1 for 72 hours using qPCR. The mRNA expression was normalized based on the housekeeping gene GAPDH. ****P ≤ .001. B, Immunoblotting for BRD4 and p21 in KYSE450 after treatment with vehicle or JQ1 for 48, 72, 96 and 144 hours. GAPDH served as a loading control. C, KYSE450 cells were treated with JQ1 for 144 hours, and cellular senescence was monitored by SA‐β‐gal staining

In addition to apoptosis, JQ1 could also trigger cellular senescence.^16^, ^17^, ^18^ To confirm whether JQ1 could induce senescence in oesophageal cancer cells, KYSE450 cells were explored to JQ1 within 6 days. The expression of p21, a protein associated with the induction of cellular senescence, was assayed. As shown in Figure 3A, the mRNA levels of p21 (cyclin‐dependent kinase inhibitors) greatly up‐regulated response to 250 nm JQ1 treatment and maintained at high levels to 10 000 nmol/L; however, p27 expression was decreased with 250 nm JQ1 treatment and then remained unchanged. Immunoblotting also demonstrated that JQ1 increased p21 protein level within 6 days in KYSE450 cells (Figure 3B). KYSE450 cells treated with JQ1 became flattened and increased the ratio of cytoplasm to nucleus (Figure 1E), which indicated KYSE450 cells might undergo cellular senescence. SA‐β‐gal‐positive staining is a hallmark of cellular senescence. As presented in Figure 3C, treated with 125nM JQ1 for 6 days, the SA‐β‐gal‐positive cells significantly rose and increased subsequently in a dose‐dependent manner.

**Figure 4:**
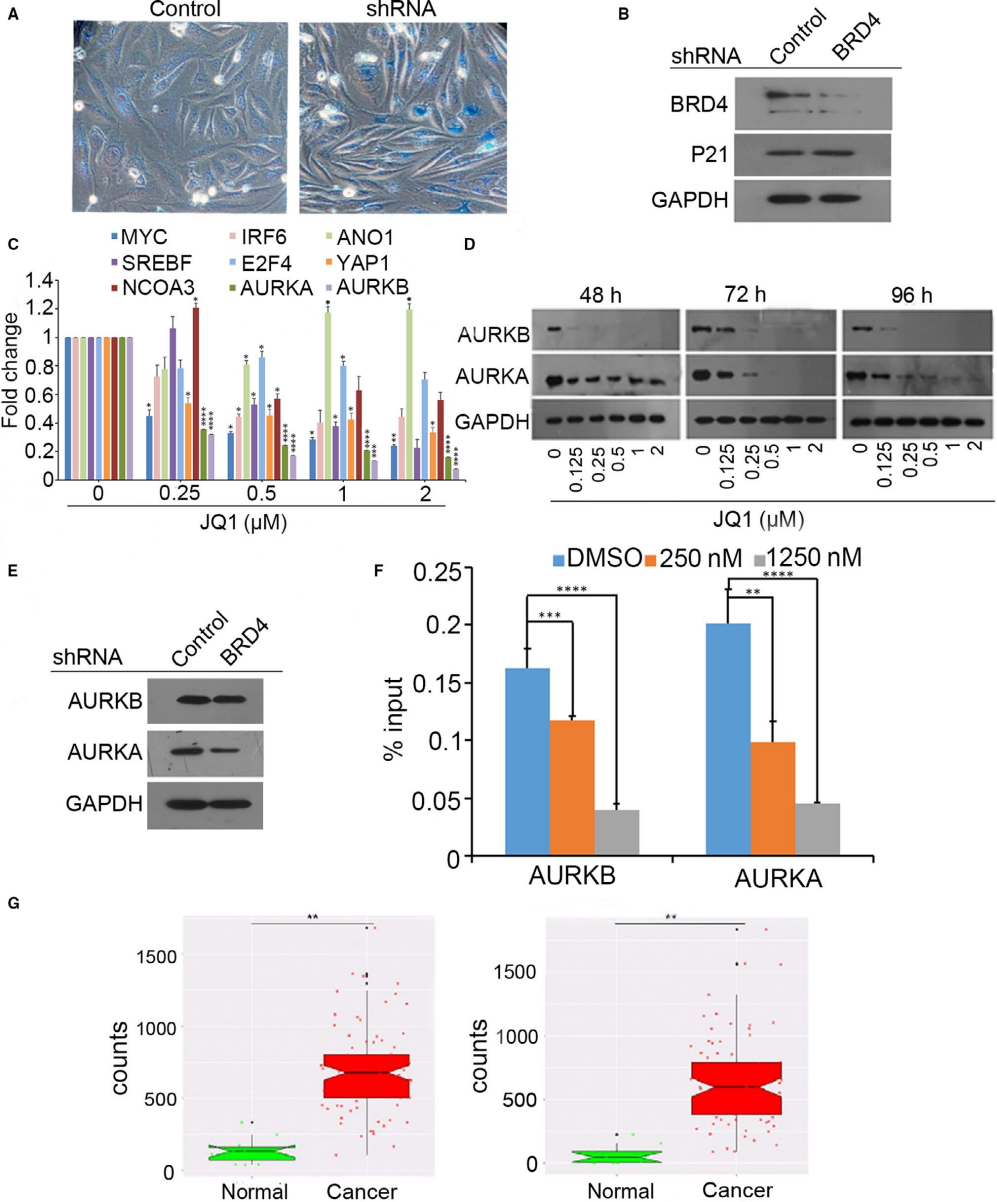
Knockdown of BRD4 induced senescence and down‐regulated AURKA and AURKB. KYSE450 cells were infected with lentivirus expressing BRD4 shRNA for 6 d. β‐Gal staining was assayed in (A) or total protein was extracted for immunoblotting in (B). C, KYSE450 cells were explored to JQ1 at different concentrations, and the transcriptional levels of potential targets were examined using qPCR. The data were presented as means ± SD. *P ≤ .05, ****P ≤ .001. D, BRD4 inhibition decreased AURKA and AURKB protein in a dose‐ and time‐dependent pattern. GAPDH served as a loading control. E, BRD4 knockdown suppressed the protein levels of AURKA and AURKB. F, Representative ChIP‐quantitative PCR assay of KYSE450 cells investigated BRD4 binding to AURKA and AURKB promoter with the presence or absence of JQ1. **P ≤ .01, ***P ≤ .005, ****P ≤ .001. G, The differential expressions of AURKA and AURKB in oesophageal cancer and normal tissues obtained from TCGA database. ** *P* ≤ .01

To further verify the roles of BRD4 in JQ1‐induced cellular senescence, KYSE450 cells were infected with lentiviruses expressing BRD4 shRNA. As shown in Figure 4A, BRD4 knockdown increased SA‐β‐gal activity resulting in elevated SA‐β‐gal‐positive staining cells. In line with senescence, the level of p21 protein was up‐regulated after BRD4 depletion (Figure 4B).

To identify the targets regulated by BRD4, we screened a couple of candidates. The results indicated aurora kinases A and B are more sensitive to BRD4 inhibition (Figure 4C). Western blot analysis also showed that AURKA and AURKB protein were time‐ and dose‐dependently decreased following JQ1 treatment (Figure 4D). Additionally, BRD4 knockdown subsequently decreased the protein levels of AURKA and AURKB (Figure 4E). Data of ChIP‐qPCR revealed that JQ1 suppressed BRD4 binding on the promoters of AURKA and AURKB genes in KYSE450 cells (Figure 4F). Finally, using the TCGA database, we determined that the expression of AURKA and AURKB in oesophageal cancer tissues is much higher than those in the normal tissues (Figure 4G). These findings suggested that the inhibition of BRD4 followed by the down‐regulation of AURKA and AURKB could induce senescence in oesophageal cancer cells, which could be used as a new therapeutic target against oesophageal cancer.

### 3.4 Blockade of AURKA and AURKB triggered senescence in KYSE450 cells

**Figure 5:**
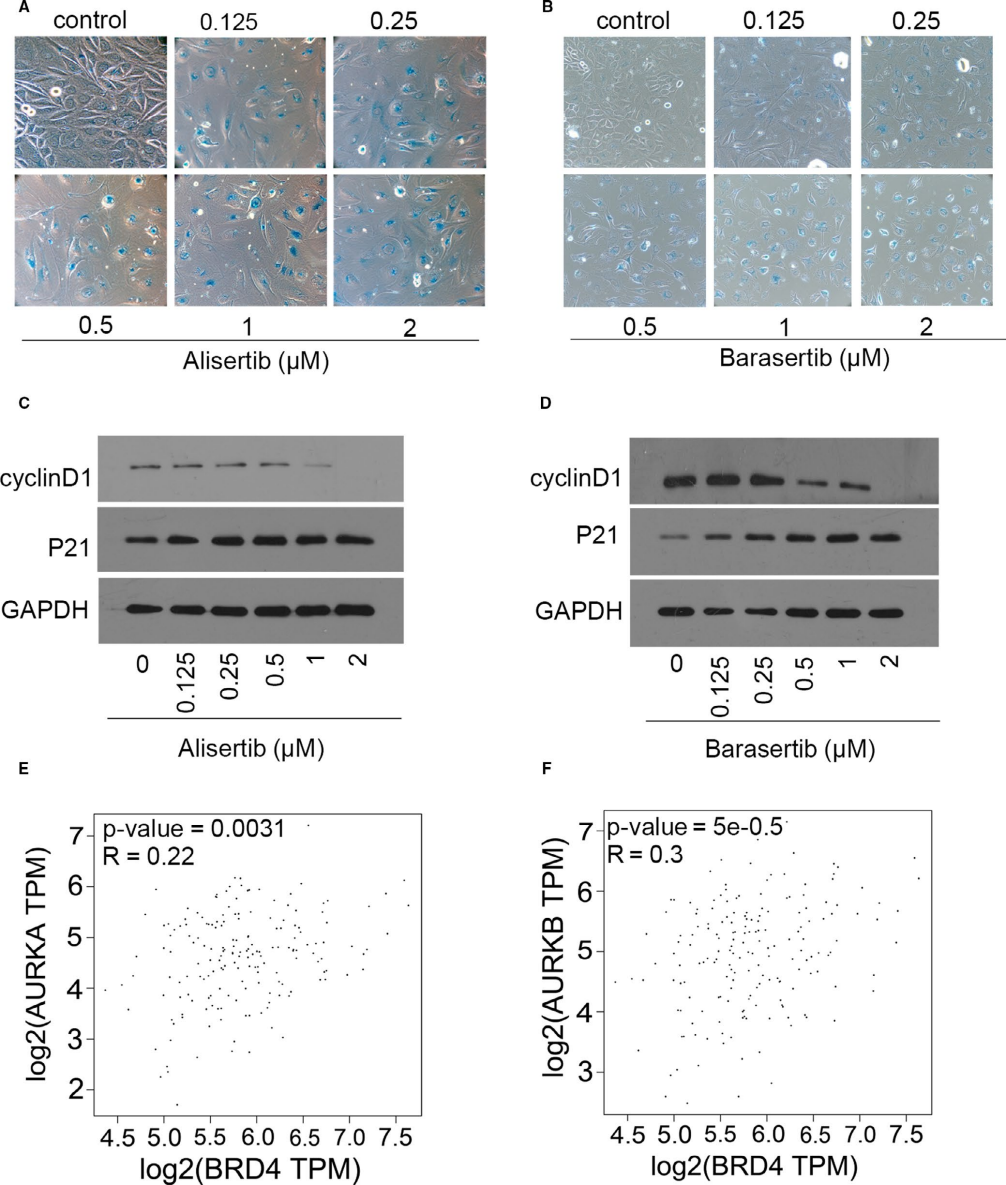
Suppression of AURKA and AURKB induced cellular senescence in oesophageal cancer cell. A, B, KYSE450 cells were treated with the AURKA or AURKB inhibitor, respectively, for 6 days, and then, cellular senescence was measured by SA‐β‐gal staining. C , D, KYSE450 cells were treated with alisertib or barasertib for 6 days, and the protein of p21 and cyclin D1 was detected using Western blotting. GAPDH served as the loading control. Scatterplots of BRD4 mRNA level vs AURKA mRNA level in (E) or AURKB mRNA level in (F) by GEPIA2 web tool. Confident intervals (CI) were 95%. *P* values and correlation coefficient are showed

To determine whether blockade of AURKA and AURKB could phenocopy JQ1‐induced cellular senescence, KYSE450 cells were explored to alisertib and barasertib, inhibitors of AURKA and AURKB, respectively. As shown in Figure 5A,B, SA‐β‐gal‐positive staining cells dramatically increased with alisertib and barasertib treatment. Moreover, the changes of p21 and cyclin D1 were consistent with those observed with BRD4 inhibition (Figure 5C,D). We analysed the correlation of AURKA or AURKB with BRD4 in oesophageal cancer using the GEPIA web‐based tool. The results showed that the expression of *AURKA* or *AURKB* is positively correlated with the levels of BRD4 mRNA (Figure 5E,F). Together, our data suggested that BRD4 inhibition induced cellular senescence by down‐regulating *AURKA* and *AURKB* in human oesophageal cancer cells.

## 4 DISCUSSION

It has been proved that BRD4 is overexpressed in many types of cancers and is a negative predictor of cancer survival.^20^, ^21^, ^22^ Inhibition of BRD4 and other proteins of the BET family by BET inhibitors was considered to be a new strategy for cancer treatment.^23^, ^24^ JQ1 is a small molecule, which is a BET bromodomain inhibitor occupying the recognition position of BRD4 competitively.^25^ Moreover, it has been found that JQ1 is a high resolution inhibitor of cancer cells and tumour growth.^26^, ^27^, ^28^ Here, we discovered anti‐proliferative roles of JQ1 on oesophageal cancer cells. KYSE450 cell line highly expressing BRD4 was most sensitive to JQ1 administration. Further investigation revealed that JQ1 arrested the cell cycle on the G1 phase but scarcely induce apoptosis. In addition, elevated p21 level and positive SA‐β‐gal staining indicated BRD4 inhibition triggered cellular senescence in KYSE450 cells. It has been proven that BETis induced cellular senescence in some cancer models.^16^, ^17^, ^18^, ^29^ However, the underlying mechanisms have not been completely explored. Inhibition of oncogene myc expression has been characterized by the canonical effects of BETis in many tumour types, but MYC‐independent manner also existed.^16^, ^24^ Our data displayed that c‐myc was decreased with JQ1 treatment in both JQ1‐sensitive and JQ1‐insensitive cell lines (Figure S1). These results indicated down‐regulation of myc was not essential for JQ1‐induced cellular senescence in oesophageal cancer. We also screened the downstream targets involved in the induction of senescence and found that AURKA and AURKB had been significantly down‐regulated treated with JQ1 treatment. JQ1 reduced BRD4 binding to the perspective promoter of AURKA and AURKB. Additionally, inhibiting the activity of either kinase by chemicals mimicked the actions of JQ1.

AURKA and AURKB are members of the aurora kinase subfamily of conserved serine/threonine kinases,^30^ which involved in cell mitosis. Aurora kinase A predominantly involved in centrosome maturation, spindle assembly and cytokinesis. However, aurora kinase B exhibited diverse function in mitotic checkpoint based on subcellular location. It has been reported that polymorphisms of AURKA or AURKB are associated with the risk and survival of breast, oesophageal and other types of cancers^31^, ^32^ suggesting that these kinases might involve in tumorigenesis and become a valuable biomarker for responsive prediction.

In conclusion, our study explored the anti‐proliferative effect of JQ1 in oesophageal cancer cells and identified the regulatory mechanism involved in their senescence through the up‐regulation of p21 and down‐regulation of AURKA and AURKB. These studies have not only provided new insights into the proliferation of BRD4‐mediated cancer cells, but also identified potential new targets, which can be used for the treatment of oesophageal cancer.

## CONFLICT OF INTEREST

The author states that there are no conflicts of interest.

## AUTHOR CONTRIBUTION

**jianling Xu:** Data curation (lead); Formal analysis (lead); Investigation (lead); Validation (equal); Writing‐original draft (lead). **yajiao yuan:** Data curation (supporting); Investigation (supporting). **jiao lv:** Data curation (supporting). **di qi:** Data curation (supporting). **mengdi wu:** Data curation (supporting). **jing lan:** Data curation (supporting). **shengnan liu:** Data curation (supporting). **hanming jiang:** Data curation (equal); Project administration (equal); Validation (equal); Writing‐review & editing (equal). **jing zhai:** Conceptualization (lead); Funding acquisition (lead); Resources (equal); Supervision (lead). **yong yang:** Funding acquisition (equal).

## Supporting information

Figure S1
