# Supplementary figures and images for: Inhibition of BRD4 triggers cellular senescence through suppressing aurora kinases in oesophageal cancer cells
Source: J Cell Mol Med. 2020 Sep 20;24(22):13036–45. doi: 10.1111/jcmm.15901 (PMC7701500; doi:10.1111/jcmm.15901)

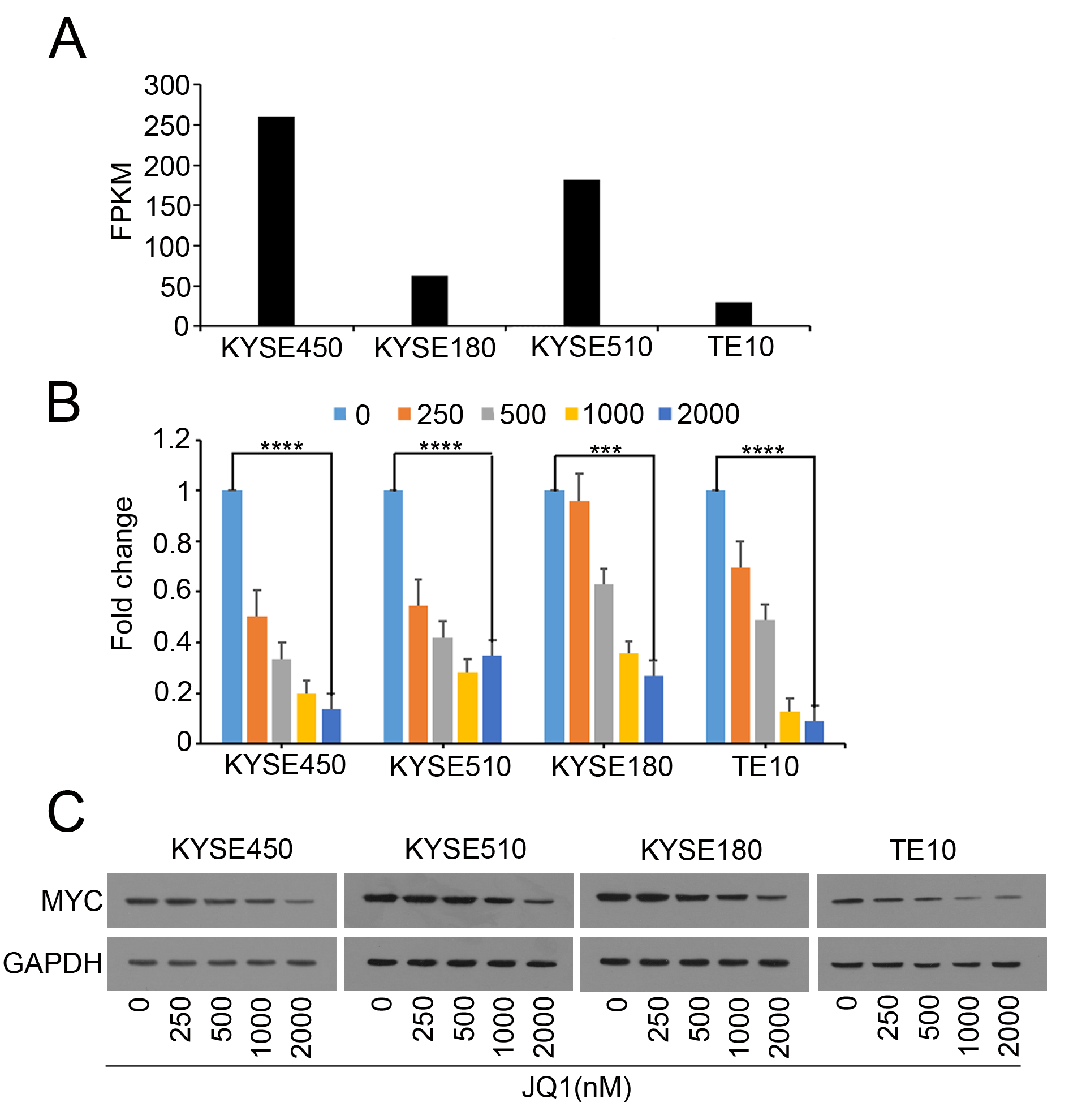

Supplement: Supplementary file 1 — Figure S1 [file JCMM-24-13036-s001.tif]
